# Curcumin inhibits breast cancer stem cell migration by amplifying the E-cadherin/β-catenin negative feedback loop

**DOI:** 10.1186/scrt506

**Published:** 2014-10-14

**Authors:** Shravanti Mukherjee, Minakshi Mazumdar, Samik Chakraborty, Argha Manna, Shilpi Saha, Poulami Khan, Pushpak Bhattacharjee, Deblina Guha, Arghya Adhikary, Sanhita Mukhjerjee, Tanya Das

**Affiliations:** Division of Molecular Medicine, Bose Institute, P-1/12, Calcutta Improvement Trust Scheme VII M, Kolkata, 700054 West Bengal India; Centre for Research in Nanoscience and Nanotechnology, University of Calcutta, Technology Campus, JD-2, Salt Lake City, Sector III, Kolkata, 700098 West Bengal India; Department of Physiology, Bankura Sammilani Medical College, Kenduadihi, Bankura, West Bengal 722101 India

## Abstract

**Introduction:**

The existence of cancer stem cells (CSCs) has been associated with tumor initiation, therapy resistance, tumor relapse, angiogenesis, and metastasis. Curcumin, a plant ployphenol, has several anti-tumor effects and has been shown to target CSCs. Here, we aimed at evaluating (i) the mechanisms underlying the aggravated migration potential of breast CSCs (bCSCs) and (ii) the effects of curcumin in modulating the same.

**Methods:**

The migratory behavior of MCF-7 bCSCs was assessed by using cell adhesion, spreading, transwell migration, and three-dimensional invasion assays. Stem cell characteristics were studied by using flow cytometry. The effects of curcumin on bCSCs were deciphered by cell viability assay, Western blotting, confocal microscopy, and small interfering RNA (siRNA)-mediated gene silencing. Evaluations of samples of patients with breast cancer were performed by using immunohistochemistry and flow cytometry.

**Results:**

Here, we report that bCSCs are endowed with aggravated migration property due to the inherent suppression of the tumor suppressor, E-cadherin, which is restored by curcumin. A search for the underlying mechanism revealed that, in bCSCs, higher nuclear translocation of beta-catenin (i) decreases E-cadherin/beta-catenin complex formation and membrane retention of beta-catenin, (ii) upregulates the expression of its epithelial-mesenchymal transition (EMT)-promoting target genes (including *Slug*), and thereby (iii) downregulates E-cadherin transcription to subsequently promote EMT and migration of these bCSCs. In contrast, curcumin inhibits beta-catenin nuclear translocation, thus impeding trans-activation of Slug. As a consequence, E-cadherin expression is restored, thereby increasing E-cadherin/beta-catenin complex formation and cytosolic retention of more beta-catenin to finally suppress EMT and migration of bCSCs.

**Conclusions:**

Cumulatively, our findings disclose that curcumin inhibits bCSC migration by amplifying E-cadherin/beta-catenin negative feedback loop.

**Electronic supplementary material:**

The online version of this article (doi:10.1186/scrt506) contains supplementary material, which is available to authorized users.

## Introduction

Breast cancer is the most common form of cancer diagnosed in women. In 2013, breast cancer accounted for 29% of all new cancer cases and 14% of all cancer deaths among women worldwide [1]. Breast cancer-related mortality is associated with the development of metastatic potential of the primary tumor [2]. Given this high rate of incidence and mortality, it is critical to understand the mechanisms behind metastasis and identify new targets for therapy. For the last few decades, various modalities of cancer therapy were being investigated. But the disease has remained unconquered, largely because of its invasive nature.

Amidst the research efforts to better understand cancer progression, there has been increasing evidence that hints at a role for a subpopulation of tumorigenic cancer cells, termed cancer stem cells (CSCs), in metastasis formation [3]. CSCs are characterized by their preferential ability to initiate and propagate tumor growth and their selective capacity for self-renewal and differentiation into less tumorigenic cancer cells [4]. There are reports which demonstrate that CSCs are enriched among circulating tumor cells in the peripheral blood of patients with breast cancer [5]. Moreover, recent studies show that epithelial-mesenchymal transition (EMT), an early step of tumor cell migration, can induce differentiated cancer cells into a CSC-like state [6]. These observations have established a functional link between CSCs and EMT and suggest that CSCs may underlie local and distant metastases by acquiring mesenchymal features which would greatly facilitate systemic dissemination from the primary tumor mass [7]. Taken together, these studies suggest that CSCs may be a critical factor in the metastatic cascade. Now, the incurability of the malignancy of the disease raises the question of whether conventional anti-cancer therapies target the correct cells since the actual culprits appear to be evasive of current treatment modalities.

Studies focusing on the early steps in the metastatic cascade, such as EMT and altered cell adhesion and motility, have demonstrated that aggressive cancer progression is correlated with the loss of epithelial characteristics and the gain of migratory and mesenchymal phenotype [8], for which downregulation of E-cadherin is a fundamental event [9]. A transcriptional consequence of the presence of E-cadherin in epithelial cells can be inferred from the normal association of E-cadherin with β-catenin in adherens junctions. This association prevents β-catenin transfer to the nucleus and impedes its role as a transcriptional activator, which occurs through its interaction mainly with the TCF (T-cell factor)-LEF (lymphoid enhancer factor) family of transcription factors but also with other DNA-binding proteins [10]. Accordingly, the involvement of β-catenin signaling in EMTs during tumor invasion has been established [11]. Aberrant expression of β-catenin has been reported to induce malignant pathways in normal cells [12]. In fact, β-catenin acts as an oncogene and modulates transcription of genes to drive cancer initiation, progression, survival, and relapse [12]. All of the existing information regarding abnormal expression and function of β-catenin in cancer makes it a putative drug target [12] since its targeting will negatively affect both tumor metastasis and stem cell maintenance. Transcriptional target genes of β-catenin involve several EMT-promoting genes, including Slug. Expression of Slug has been shown to be associated with breast tumor recurrence and metastasis [13–15]. Pro-migratory transcription factor Slug (EMT-TF), which can repress E-cadherin, triggers the steps of desmosomal disruption, cell spreading, and partial separation at cell-cell borders, which comprise the first and necessary phase of the EMT process [16].

Recently, the use of natural phytochemicals to impede tumor metastasis *via* multiple targets that regulate the migration potential of tumor cells has gained immense importance [17]. In this regard, curcumin, a dietary polyphenol, has been studied extensively as a chemopreventive agent in a variety of cancers, including those of the breast, liver, prostate, hematological, gastrointestinal, and colorectal cancers, and as an inhibitor of metastasis [18]. In a recent report, curcumin was shown to selectively inhibit the growth and self-renewal of breast CSCs (bCSCs) [19]. However, there are no reports regarding the contribution of curcumin in bCSC migration.

The present study describes (i) the mechanisms governing the augmented migration potential of bCSCs, which (ii) possibly associates with tumor aggressiveness and is largely attributable to the inherent downregulation of the anti-migratory tumor suppressor protein, E-cadherin, in bCSCs, and (iii) the role of curcumin in modulating the same. A search for the upstream mechanism revealed higher nuclear translocation and transcriptional activity of β-catenin resulting from disruption of E-cadherin/β-catenin complex formation in bCSCs in comparison with non-stem tumor cells. Upregulation of nuclear β-catenin resulted in the augmentation of *Slug* gene expression that, in turn, repressed E-cadherin expression. In contrast, exposure to curcumin inhibited the nuclear translocation of β-catenin, thereby hampering the activation of its EMT-promoting target genes, including *Slug*. Resultant upregulation of E-cadherin led to increase in E-cadherin/β-catenin complex formation, which further inhibited nuclear translocation of β-catenin. As a consequence, the E-cadherin/β-catenin negative feedback loop was amplified upon curcumin exposure, which reportedly inhibits EMT on one hand and promotes cell-cell adherens junction formation on the other. These results suggest that curcumin-mediated inhibition of bCSC migration may be a possible way for achieving CSC-targeted therapy to better fight invasive breast cancers.

## Materials and methods

### Primary tissue culture

Primary human breast cancer tissue samples used in this study were obtained with informed consent from all patients from Department of Surgery, Bankura Sammilani Medical College, Bankura, India, in accordance with the Institutional Human Ethics Committee (approval letter CNMC/ETHI/162/P/2010), and the associated research and analyses were done at Bose Institute, Kolkata, India, in compliance with the Bose Institute Human Ethics Committee (approval letter BIHEC/2010-11/11). These tumors were exclusively primary-site cancers that had not been treated with either chemotherapy or radiation. The selected cases consisted of three primary breast cancer patients of each group. The specimens were washed with phosphate-buffered saline (PBS), cut into small pieces (5 × 5 mm in size), and immersed in a mixture of colloagenase (10%; Calbiochem, now part of EMD Biosciences, Inc., San Diego, CA, USA) and hyaluronidase (0.5 mg/mL; Calbiochem) for 12 to 16 hours at 37°C on orbital shaker. The contents were centrifuged at 80 *g* for 30 seconds at room temperature. The supernatant, comprising mammary fibroblasts, was discarded, and to the pellet pre-warmed 0.125% trypsin-EDTA was added. The mixture was gently pipetted and kept for 30 minutes at 37°C. Finally, the pellet obtained was washed with cold Hanks’ buffer saline with 2% fetal bovine serum and centrifuged at 450 *g* for 5 minutes at room temperature. The single cells were seeded on poly-L lysine-coated dishes and cultured in medium containing growth factors, 0.1 ng/mL human recombinant epidermal growth factor, 5 μg/mL insulin, 0.5 μg/mL hydrocortisone, 50 μg/mL gentamycin, 50 ng/mL amphotericin-B, and 15 μg/mL bovine pituitary extract at 37°C. Medium was replaced every 4 days, and passages were done when the cells reached 80% confluence [20].

### Cell culture and treatment

Human breast cancer cell lines MCF-7 and T47D were obtained from the National Centre for Cell Science (Pune, India). The cells were routinely maintained in complete Dulbecco’s modified Eagle’s medium (DMEM) supplemented with 10% heat-inactivated fetal bovine serum (FBS), penicillin (100 units/mL), and streptomycin (100 l g/mL) at 37°C in a humidified incubator containing 5% CO_2_. Cells were allowed to reach confluency before use. Cells were maintained in an exponential growth phase for all experiments. All cells were re-plated in fresh complete serum-free medium for 24 hours prior to the experiments. Viable cell numbers were determined by Trypan blue dye exclusion test [21]. Cells were treated with different doses (5, 10, 15, and 20 μM) of curcumin (Sigma-Aldrich, St. Louis, MO, USA) for 24 hours to select the optimum non-apoptotic dose of curcumin (15 μm) which significantly abrogates migration potential of bCSCs. An equivalent amount of carrier (dimethyl sulfoxide) was added to untreated/control cells. To rule out cell proliferation, all migration assays were performed in the presence of 10 μg/mL mitomycin C.

### Mammosphere culture

For mammosphere culture, MCF-7/T47D cells were seeded at 2.5 × 10^4^ cells per well in sixwell Ultralow Adherence plates (Corning Inc., Corning, NY, USA) in DMEM/F12 with 5 μg/mL bovine insulin (Sigma-Aldrich), 20 ng/mL recombinant epidermal growth factor, 20 ng/mL basic fibroblast growth factor, B27 supplement (BD Biosciences, San Jose, CA, USA), and 0.4% bovine serum albumin (BSA) as previously described [22]. Primary/1° and secondary/2° mammosphere formation was achieved by using weekly trypsinization and dissociation followed by reseeding in mammosphere media at 2.5 × 10^4^ cells per well into Ultralow Adherence sixwell plates.

### Cell viability assay

Cell viability assay was performed by using Trypan blue dye exclusion assay. Mammospheres were treated with different doses of curcumin for 24 hours. Thereafter, the numbers of viable cells were counted by Trypan blue dye exclusion by using a hemocytometer. The results were expressed as percentage relative to the control cells.

### Flow cytometry

Expression of human bCSC markers CD44 and CD24 were analyzed by flow cytometric study in different stages of breast cancer tissue as well as in MCF-7/T47D cells and primary and secondary mammospheres by using CD44-FITC and CD24-PE antibodies (BD Biosciences). bCSCs were flow-cytometrically sorted from primary breast tumors on the basis of the cell surface phenotype CD44^+^/CD24^-/low^. De-differentiation, drug resistance, and stemness phenomena were quantified flow-cytometrically by measuring mean fluorescence intensities of de-differentiation markers Oct-4-PerCP-Cy5.5, Nanog-PE, and Sox-2-Alexa Fluor-647; drug-resistance markers MRP1-FITC, ABCG2-PE, and ALDH1-FITC (BD Biosciences); and epithelial markers cytokeratin-18-PE and cytokeratin-19-PE (Santa Cruz Biotechnology, Inc., Santa Cruz, CA, USA). Expression levels of E-cadherin, β-catenin, and Slug (Santa Cruz Biotechnology, Inc.) were determined with respective primary antibodies conjugated with PE as previously described [23].

### Immunofluorescence

For immunofluorescence, cells were grown on sterile glass coverslips at 37°C for 24 hours. Cells after treatment were washed briefly with PBS and fixed with 4% formaldehyde for 20 minutes at 37°C and permeabilized with Triton X100 (for intracellular protein expression analysis). Thereafter, cells were blocked for 2 hours in a blocking buffer (10% BSA in PBS) and incubated for another hour in PBS with 1.5% BSA containing anti-CD44/CD24/E-cadherin/β-catenin/phospho-FAK antibody (Santa Cruz Biotechnology, Inc.). After washing in PBS, cells were incubated with FITC/PE-conjugated secondary antibodies in PBS with 1.5% BSA for 45 minutes at 37°C in the dark. 4′-6-diamidino-2-phenylindole (DAPI) was used for nuclear staining. Coverslips were washed with PBS and mounted on microscopy glass slides with 90% glycerol in PBS. Images were acquired by using a confocal microscope (Carl Zeiss, Jena, Germany) [21].

### Wound-healing assay

To determine the expression of bCSC markers in the migrating versus non-migrating fraction of MCF-7 cells, bi-directional wound-healing assay was performed. Briefly, cells were grown to confluency on sterile glass coverslips, after which a sterile 10-μL tip was used to scratch the monolayer of cells to form a bi-directional wound. Cells were allowed to migrate for 24 hours and then the coverslips were used for immunofluorescence staining.

### Transwell migration assay

Transwell migration assay was performed by using 8.0-μm cell culture inserts (BD Biosciences) to test the migratory ability of primary breast cancer cells, MCF-7/T47D cells, and mammosphere-forming cells. Cells were seeded at 2.5 × 10^5^ cells per well in serum-free DMEM in the upper chamber of 12-well plates and allowed to migrate for 8 hours toward DMEM containing 10% FBS in the lower chamber. After 8 hours, the cells in the upper chamber were removed with a cotton swab and the migrated cells in the lower surface of the membrane were fixed and stained with giemsa or the migrated fraction of 2° mammospheres were collected from the under-surface of the membranes after 24-hour migration assay for flow cytometry. Images were acquired with a brightfield microscope (Leica, Wetzlar, Germany) at 20× magnification. To quantify migratory cells, three independent fields were analyzed by using ImageJ software (National Institutes of Health, Bethesda, MD, USA). Migration was expressed as percentage of cells migrated. For the same, the percentage of cells that migrated in the control set of each relevant experiment was taken as 100%.

### Cell adhesion assay

For evaluating cell adhesion property, cells were trypsinized by using trypsin-EDTA and resuspended in DMEM at a density of 0.8 × 10^6^ cells per milliliter. These cell suspensions were allowed to recover from the trypsinization for 1 hour at 37°C in a humidified incubator containing 5% CO_2_. They were mixed gently every 15 minutes during this hour of conditioning. After every 15 minutes of incubation, the dishes were removed from the incubator, and the medium containing unattached cells was removed. Images were acquired with an Olympus BX700 inverted microscope (Olympus, Tokyo, Japan) at 20× magnification. To quantify cell adhesion, the number of unattached cells at 1 hour was determined by counting three independent fields. Attachment (at 1 hour) was expressed as percentage of cells adhered, and the percentage of the control set of each relevant experiment was taken as 100%.

### Cell spreading assay

Spreading of the attached cells was monitored. At various time intervals (for every 30 minutes up to 3 hours), cells were imaged by using an Olympus BX700 inverted microscope (Olympus). Images of multiple fields were captured from each experimental set at 40× magnification. From the phase-contrast images, individual cell boundaries were marked with the free-hand tool of ImageJ, and the area within the closed boundary of each cell was quantified by using the analysis tool of ImageJ. Cell spreading (at 3 hours) was expressed as mean circularity of the cells. As confirmation assay for cell adhesion and spreading, MCF-7 cells and 2° mammosphere cells were plated on fibronectin (50 μg/mL)-coated surface, and focal adhesions were stained and quantified by immunofluorescence staining for phospho-FAK. In fact, phospho-FAK-enriched clusters at lamellipodia were considered as focal adhesion complex. Focal adhesion segmentation and size measurement were done by using ImageJ software.

### Three-dimensional invasion assay

Three-dimensional (3D) invasion assay of mammospheres was performed in 96-well plates. Each well was first coated with 80 μL matrigel (BD Biosciences) in 3:1 ratio with complete DMEM. Mammospheres with or without curcumin/small interfering RNA (siRNA)/short hairpin RNA (shRNA)/cDNA treatment were mixed with matrigel (6:1) and added to the previously coated wells. Thereafter, the mammospheres were allowed to invade for 48 hours. Images were photographed by using an Olympus BX700 inverted microscope (Olympus) at 20× magnification. Data were analyzed by using ImageJ software as area invaded and were expressed as percentage relative to the control set, the value of which was taken as 100%.

### Western blotting and co-immunoprecipitation

To obtain whole cell lysates, cells were homogenized in buffer (20 mM Hepes, pH 7.5, 10 mM KCl, 1.5 mM MgCl_2_, 1 mM Na-EDTA, 1 mM Na-EGTA, and 1 mM DTT). All buffers were supplemented with protease and phosphatase inhibitor cocktail [24, 25]. Protein concentrations were estimated by using Lowry’s method. An equal amount of protein (50 μg) was loaded for Western blotting. For direct Western blot analysis, the cell lysates or the particular fractions were separated by SDS-PAGE, transferred to polyvinylidene difluoride membrane (Millipore, Darmstadt, Germany), and probed with specific antibodies like anti-E-cadherin, anti-β-catenin, anti-histone H1, anti-cyclin-D1, anti-c-myc, anti-slug, anti-vimentin, anti-MMP-2, anti-MMP-9, anti-twist, anti-Snail, and anti-α-Actin (Santa Cruz Biotechnology, Inc.). The protein of interest was visualized by chemiluminescence (GE Biosciences, Piscataway, NJ, USA). To study the interaction between E-cadherin and β-catenin, β-catenin immunocomplex from whole cell lysate was purified by using β-catenin antibody and protein A-Sepharose beads (Invitrogen, Frederick, MD, USA). The immunopurified protein was immunoblotted with E-cadherin antibody. The protein of interest was visualized by chemi-luminescence. Equivalent protein loading was verified by using anti-α-actin/Histone H1 antibody (Santa Cruz Biotechnology, Inc.) [26].

### Reverse transcription-polymerase chain reaction assay

Two micrograms of the total RNA, extracted from cells with TRIzol reagent (Invitrogen, Carlsbad, CA, USA), was reverse-transcribed and subjected to polymerase chain reaction (PCR) with enzymes and reagents of the RTplusPCR system (Eppendorf, Hamburg, Germany) by using GeneAmpPCR 2720 (Applied Biosystems, Foster City, CA, USA). The cDNAs were amplified with specific primers for E-cadherin (forward-CACCTGGAGAGAGGCCATGT, reverse-TGGGAAACAT-GAGCAGCTCT) and glyceraldehyde 3-phosphate dehydrogenase (GAPDH) (forward-CGT-ATTGGGCGCCTGGTCAC, reverse-ATGATGACCCTTT-TGGCTCC).

### Plasmid and small interfering RNA/short hairpin RNA transfections

Cells were transfected separately with 300 pmol of E-cadherin shRNA (Addgene, Cambridge, MA, USA) or Slug siRNA (Santa Cruz Biotechnology, Inc.) by using Lipofectamine 2000 (Invitrogen). The levels of respective proteins were estimated by Western blotting. The Slug cDNA (Addgene) plasmid was used for overexpression studies. The Slug cDNA clone was introduced in cells by using Lipofectamine 2000. Stably expressing clones were isolated by limiting dilution and selection with G418 sulphate (Cellgro, a brand of Mediatech, Inc., Manassas, VA, USA) at a concentration of 400 μg/mL, and cells surviving this treatment were cloned and screened by Western blot analysis with specific antibodies.

### Immunohistochemistry

Tissues were dissected out; fixed in Bouin’s fixative overnight; cryoprotected in 10% (2 hours), 20% (2 hours), and 30% (overnight) sucrose solution in PBS at 4°C; and frozen with expanding CO_2_, and serial sections were cut on a cryostat (CM1850; Leica) at 15-μm thickness. The tissue sections were washed in PBS (pH 7.45) for 15 minutes and treated with 1% BSA in PBS containing 0.1% Triton X-100. Sections were incubated overnight at 25°C in a humid atmosphere with primary antibodies against E-cadherin (1:100; Santa Cruz Biotechnology, Inc.) diluted in PBS containing and 1% BSA. Sections were rinsed in PBS for 10 minutes and incubated with biotinylated anti-mouse IgG (Sigma-Aldrich; 1:100) for 1 hour, followed by ExtrAvidin-peroxidase conjugate (Sigma-Aldrich; 1:100) for 40 minutes. 3-Amino-9-ethyl carbazole was used as chromogen (Sigma-Aldrich; 1:100) to visualize the reaction product. Thereafter, sections were counterstained with hematoxylin (1:1; Himedia, Mumbai, India). Finally, sections were washed in distilled water and mounted in glycerol gelatin. Images were acquired with a brightfield microscope (Leica) at 10× magnification.

### Statistical analysis

Values are shown as standard error of mean unless otherwise indicated. Comparison of multiple experimental groups was performed by two-way analysis-of-variance test. Data were analyzed; when appropriate, significance of the differences between mean values was determined by a Student’s *t* test. Results were considered significant at a *P* value of not more than 0.05.

## Results

### Breast cancer stem cells, being highly migratory, are linked with aggressiveness of the disease

**Figure 1:**
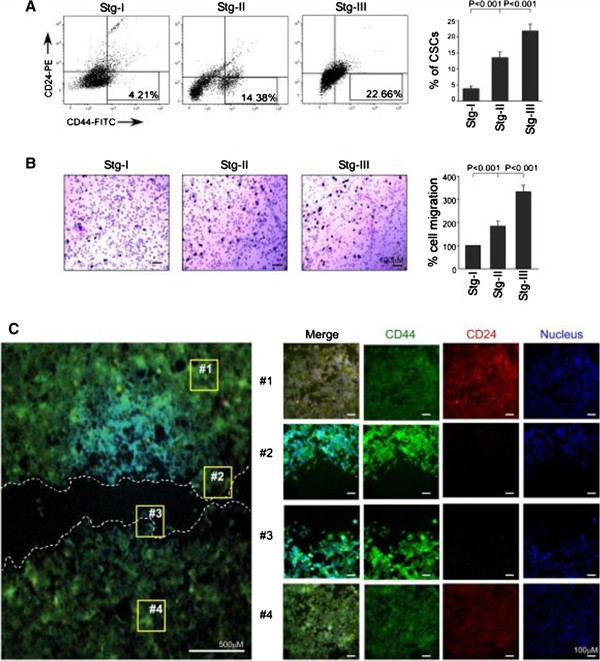
**Breast cancer stem cells (CSCs) are highly migratory and are correlated with aggressiveness of the disease. (A)** The percentage content of breast CSCs (CD44^+^/CD24^-/low^) in different stages of breast cancer was determined by flow cytometry and represented graphically (right panel). The left panel depicts representative flow cytometry data. **(B)** Migration of primary breast cancer cells of different stages was evaluated by using transwell migration assay. Cells that had migrated to the lower surface of the 8.0-μm membrane were stained with Giemsa stain, counted, and represented graphically (right panel). The left panel shows brightfield images of migration assay of different breast cancer stages. **(C)** Expression of CSC markers (CD44^+^/CD24^-/low^) was visualized by immunofluorescence in the migrating front and non-migrating pool of MCF-7 cells after 24-hour wound-healing assay. Data are presented as mean ± standard error of mean or representative of three independent experiments.

To determine whether CSCs are linked with tumor aggressiveness or malignancy, we performed flow cytometric analyses of bCSC markers CD44^+^/CD24^-/low^ in patient-derived tumor samples of different stages. We also tested the migratory potentials of these primary cells of different stages of cancer by performing transwell migration assay. Interestingly, along with the gradual increase in percentage cell migration, that is, 188.67% ± 9.33% (*P* <0.001) and 337.33% ± 20.34% (*P* <0.001) in stages II and III, respectively, as compared with stage I, which was taken as 100%, there was increase in the CSC content too, that is, 4.2% ± 0.40%, 14.17% ± 0.75%, and 21.13% ± 1.80% CSCs (*P* <0.001) in stages I, II, and III, respectively (Figure 1A and B), indicating that the CSC population is proportionally related with breast cancer migration. In a parallel experimental set using the razor-wound migration assay method, human breast cancer cell line MCF-7 furnished higher expression of CSC-markers (that is, CD44^+^/CD24^-/low^) in the migrating population as compared with the non-migrating fraction of cells as evident from our confocal data (Figure 1C). In line with an earlier report [27], these results revealed that the increase in expression of CSC markers selects for breast cancer cells with enhanced malignant and metastatic ability.

**Figure 2:**
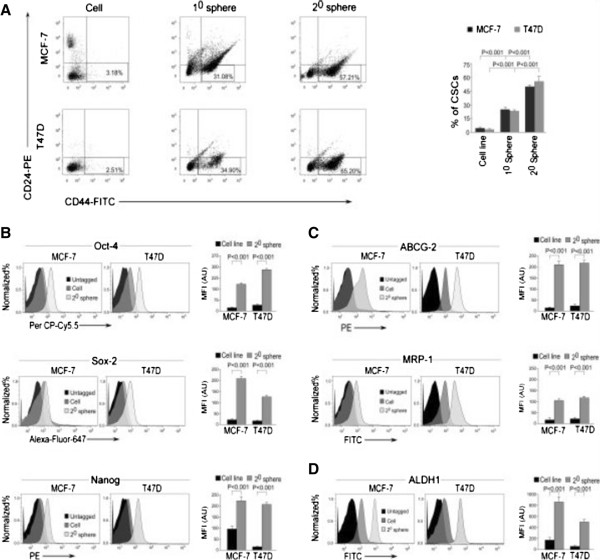
**Relative quantification of breast cancer stem cells in MCF-7 and T47D cell lines and their mammospheres along with their characterization for stemness properties. (A)** The percentage content of breast cancer stem cells (CD44^+^/CD24^-/low^) in MCF-7 and T47D cells, MCF-7/T47D-derived primary/1° and secondary/2° mammospheres, were determined by flow cytometry and represented graphically (right panel). The left panel depicts representative flow cytometry data. **(B-D)** Graphical representation of relative mean fluorescence intensities (MFIs) in arbitrary units (AU) of de-differentiation markers Oct-4, Sox-2, and Nanog; drug-resistance markers ABCG2 and MRP1; and stemness-related enzyme ALDH1 in MCF-7 and T47D cell lines, along with their respective 2° mammospheres as determined by flow cytometry (right panels). The left panels depict representative flow cytometric histogram overlay data. Data are presented as mean ± standard error of mean or representative of three independent experiments.

**Figure 3:**
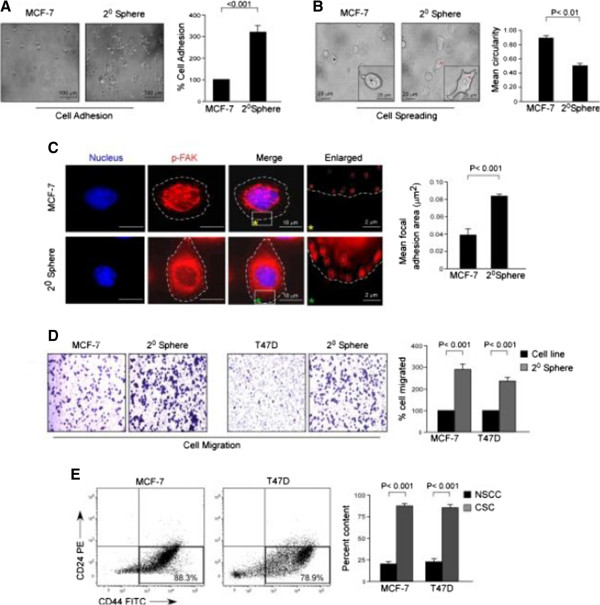
**Breast cancer stem cell (CSC)-enriched mammospheres exhibit highly aggravated migratory properties. (A, B)** Representative phase-contrast images of cell adhesion and spreading assays of MCF-7 and 2° mammosphere-forming cells (left panels). The right panels demonstrate relative quantification of the data. **(C)** Confocal images showing focal adhesions in MCF-7 and 2° mammosphere-forming cells, stained with phospho-FAK (PE) (red) and nuclear stain 4′-6-diamidino-2-phenylindole (DAPI) (left panel). The right panel illustrates relative quantification data of mean focal adhesion area. **(D)** Representative brightfield images of transwell migration assays of MCF-7 and T47D cells and their respective 2° mammosphere-forming cells (left and middle panels). The right panel demonstrates relative quantification of the data graphically. **(E)** The percentage content of breast CSCs (CD44^+^/CD24^-/low^) in the migrated fractions of 2° mammospheres of MCF-7 and T47D cell lines as compared with non-stem cancer cells (NSCCs) was determined by flow cytometry and represented graphically (right panel). The left panel depicts representative flow cytometry data. Data are presented as mean ± standard error of mean or representative of three independent experiments.

Our next attempt was to evaluate the migratory properties of bCSCs as compared with the non-stem tumor population. For the same, the percentage CSC content of MCF-7 and T47D, as well as of primary/1° and secondary/2° mammospheres generated from these two cell lines, was elucidated by using flow cytometry for the bCSC phenotype, CD44^+^CD24^-/low^. Results of Figure 2A depict the presence of 4.3% ± 0.70% CSCs in MCF-7, 26.72% ± 2.40% in its 1° mammosphere, and 52.17% ± 2.86% in 2° mammosphere (*P* <0.001), and the percentages were 2.04% ± 0.49% in T47D, 25.44% ± 2.32% in its 1° mammosphere, and 57.47% ± 4.02% in 2° mammosphere (*P* <0.001). Since 2° mammospheres were found to be highly CSC-enriched, all of our later studies for bCSCs were performed with 2° mammospheres. Next, we re-confirmed the presence of significantly higher stemness properties in the mammospheres as compared with MCF-7/T47D cells by comparing the expression levels of pleuripotency markers, Oct-4, Sox-2, and Nanog (Figure 2B); de-differentiation and drug-resistance markers, ABCG2 and MRP1 (Figure 2C); and ALDH1 (Figure 2D). After the presence of higher stemness and CSC enrichment in the mammospheres of both the breast cancer cell lines MCF-7 and T47D was validated, all of our later experiments were performed with mammospheres of MCF-7 cells while re-confirming the key experiments in mammospheres of T47D cells. Next, we compared the migration efficiency of mammospheres with MCF-7 cells. Interestingly, these bCSC-enriched mammospheres were found to be highly migratory as compared with MCF-7 cells within the same time frame. Briefly, mammosphere-forming cells exhibited higher adhesion property than MCF-7 cells; that is, 316% ± 18.19% mammosphere-forming cells were adhered as compared with MCF-7 cells (100%) (*P* <0.001; Figure 3A). Similarly, mammosphere cells demonstrated lesser circularity (0.503 ± 0.04 mean circularity) than MCF-7 cells (0.873 ± 0.04 mean circularity), thereby depicting higher mesenchymal and migration properties of mammospheres (*P* <0.01; Figure 3B). At this juncture, for more robust assessment of adhesion, we quantified the size of phospho-FAK-enriched focal adhesion area from the lammellipodia of MCF-7 and its 2° mammosphere-forming cells. Our results showed that the mean focal adhesion area of mammosphere-forming cells was significantly higher (*P* <0.001) as compared with that of MCF-7 cells (Figure 3C). Even in transwell migration assay, the percentage migration of mammosphere cells (293.67% ± 9.56%) was higher than that of MCF-7 cells (taken as 100%) (*P* <0.001; Figure 3D). Results of Figure 3D validated the findings of transwell migration assay in the T47D cell line and its mammospheres.

At this stage, we considered the possibility that, since the mammosphere is a heterogeneous population of cells consisting of both CSCs and non-stem cancer cells, the migrated population of the mammosphere might be a heterogeneous one. It therefore becomes debatable whether the aggravated migration property of mammospheres is the contribution of bCSCs or of non-stem cancer cells. To get the answer, the migrated cells of the mammospheres were collected from the under-surface of the membranes, and flow cytometric analyses were performed to characterize the migrated cells. Results of Figure 3E demonstrated that the majority of the migrating cells of the mammospheres were bCSCs for both the cell lines, that is, 83.67% ± 2.90% bCSCs for mammospheres of MCF-7 (*P* <0.001) and 80.33% ± 3.48% (*P* <0.001) bCSCs for mammospheres of T47D. These results validate that bCSCs are endowed with aggravated migration potential as compared with the rest of the non-stem tumor population.

### bCSCs exhibit enhanced migration potential through suppression of the EMT marker, E-cadherin

**Figure 4:**
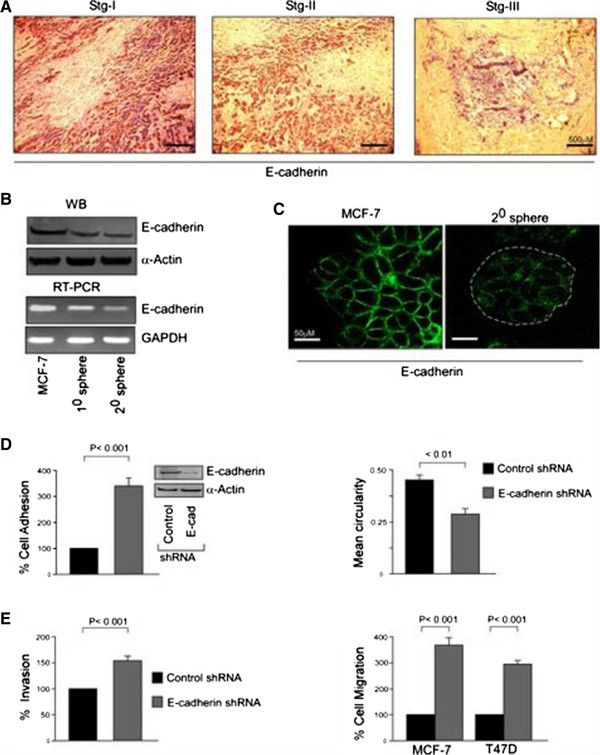
**The augmented migration potential of breast cancer stem cells (bCSCs) results from the suppression of the epithelial-mesenchymal transition (EMT) marker, E-cadherin. (A)** Immunohistological staining for E-cadherin (brown color for antibody staining and counterstained with hematoxylin) of breast tumor samples. **(B)** Protein and mRNA expression profiles of E-cadherin in MCF-7 cells, 1° and 2° mammospheres, was determined by Western blotting (WB) (upper panel) and reverse transcription-polymerase chain reaction (RT-PCR) (lower panel). **(C)** Expression of E-cadherin in MCF-7 cells and 2° mammospheres was visualized by immunofluorescence. **(D)** Graphical representation of relative cell adhesion (left panel) and spreading (right panel) of MCF-7-derived 2° mammospheres with or without transfection with E-cadherin-short hairpin RNA (shRNA). The efficiency of transfection was assessed by evaluating the expression of E-cadherin through WB (inset). **(E)** A similar experimental setup was scored for three-dimensional (3D) invasion (left panel) and transwell migration (right panel) assays. Transwell migration assay was performed under similar experimental conditions in T47D-derived 2° mammospheres (right panel). α-Actin/glyceraldehyde 3-phosphate dehydrogenase (GAPDH) was used as an internal loading control. Data are presented as mean ± standard error of mean or representative of three independent experiments.

Our effort to delineate the mechanism underlying the enhanced migratory behavior of bCSCs revealed suppression of E-cadherin expression, loss of which (a hallmark of EMT) has been reported to promote tumor metastasis [28]. In fact, our immunohistochemical analyses revealed a gradual decrease in the expression levels of E-cadherin protein with increasing stages of breast cancer (Figure 4A). Results of our Western blot and reverse transcription-PCR analyses also elucidated lower protein and mRNA levels of E-cadherin in mammospheres than in MCF-7 cells (Figure 4B). The same results were obtained in our confocal analyses (Figure 4C). In our previous findings, we have shown an increase in CSC percentage with an increase in the stage of breast cancer (Figure 1A). Therefore, we postulated that probably bCSCs maintain their aggravated migration property through suppression of the E-cadherin protein expression. As a validation of this hypothesis, shRNA-mediated silencing of E-cadherin protein expression in mammospheres resulted in significant augmentation of the migratory phenotype of these mammospheres, as reflected in our cell-adhesion assay; that is, 316.67% ± 23.33% E-cadherin-silenced mammosphere cells adhered as compared with the control shRNA-transfected cells (100%) (*P* <0.001; Figure 4D, *left panel*). Similarly, E-cadherin-ablated mammospheres demonstrated augmented cell spreading as depicted by loss in mean circularity of cells: that is, 0.45 ± 0.02 and 0.27 ± 0.03 mean circularity of cells of control shRNA-transfected and E-cadherin-silenced mammospheres, respectively (*P* <0.01; Figure 4D, *right panel*). In addition, 3D invasion potential of E-cadherin-knocked-down mammospheres was also elevated (161.67% ± 7.31%) when compared with control shRNA-transfected set (100%) (*P* <0.001; Figure 4E, *left panel*). These results were finally confirmed in our transwell migration assay in which E-cadherin-shRNA-transfected mammosphere cells showed 340.67% ± 26.97% migration as compared with 100% migration of control shRNA-transfected cells (*P* <0.001; Figure 4E, *right panel*). Transwell migration assay of mammospheres of T47D cells also rendered similar results: that is, 291.67% ± 15.41% cell migration in E-cadherin-shRNA transfected mammospheres as compared with 100% cell migration in control shRNA set (*P* <0.001; Figure 4E, *right panel*). Taken together, these results validate that suppressed expression of E-cadherin is essential for maintaining accentuated migration potential of bCSCs.

### Suppression of E-cadherin expression in bCSCs is associated with greater nuclear translocation and transcriptional activity of β-catenin

**Figure 5:**
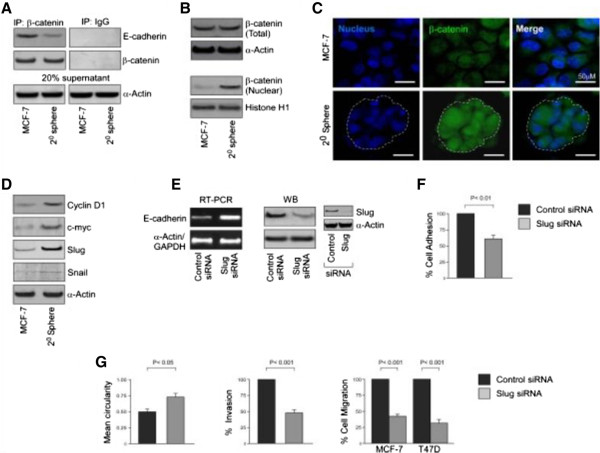
**E-cadherin suppression in breast cancer stem cells (bCSCs) is associated with greater nuclear translocation of β-catenin and subsequent trans-activation of Slug. (A)** β-catenin-associated E-cadherin was assayed by co-immunoprecipitation from cell lysates of MCF-7 and 2° mammospheres by using specific antibodies (left panel) or with normal human immunoglobulin G (IgG) as a negative control (right panel). To ensure comparable protein loading, 20% of supernatant from immunoprecipitation (IP) sample was subjected to determination of α-actin by Western blotting (WB). **(B)** WB was conducted to study the levels of total β-catenin and nuclear β-catenin in MCF-7 and 2° mammospheres for determining the nuclear translocation of β-catenin. **(C)** The relative nuclear expression of β-catenin in MCF-7 and 2° mammospheres was visualized by immunofluorescence. **(D)** WB was performed to study the expression levels of β-catenin target genes *Cyclin-D1, c-Myc, Slug* and *Snail* in MCF-7 cells and 2° mammospheres. **(E)** Protein and mRNA expression profiles of E-cadherin in 2° mammospheres of MCF-7 cells with or without transfection with Slug-short interfering RNA (siRNA) were determined by WB (right panel) and reverse transcription-polymerase chain reaction (RT-PCR) (left panel). The efficiency of transfection was assessed by evaluating the expression of Slug through WB (inset). **(F, G)** Graphical representation of relative cell adhesion, spreading, three-dimensional invasion, and transwell migration of MCF-7-derived 2° mammospheres with or without transfection with Slug siRNA. Transwell migration assay was also performed under similar experimental conditions in T47D-derived 2° mammospheres (G, right panel). α-Actin/histone H1/glyceraldehyde 3-phosphate dehydrogenase (GAPDH) was used as an internal loading control. Data are presented as mean ± standard error of mean or representative of three independent experiments.

There are several reports delineating the pro-migratory role of β-catenin protein [29, 30]. Moreover, activation of β-catenin pathway has been reported in CSCs [31]. Under normal conditions, β-catenin exists in physical association with membrane-bound E-cadherin. However, if unbound with surface E-cadherin, β-catenin becomes free to translocate to the nucleus and transcriptionally activates several pro-migratory genes necessary for EMT in association with the TCF/LEF transcription factors [12]. Results of our co-immunoprecipitation studies revealed a much lower association between E-cadherin and β-catenin proteins in mammospheres as compared with MCF-7 cells (Figure 5A). Moreover, although the total β-catenin protein level remained unaltered, a significantly higher nuclear level of the protein was observed in mammospheres than MCF-7 cells (Figure 5B). Higher nuclear localization of β-catenin in mammospheres was confirmed by confocal microscopy (Figure 5C). That the transcriptional activity of β-catenin was augmented in mammospheres was confirmed in our Western blotting data, in which greater expression of cyclin-D1, c-myc, and Slug proteins (Figure 5D), which are direct transcriptional targets of β-catenin [12], was observed. However, the expression levels of another important β-catenin transcriptional target, Snail, not only was very low in both MCF-7 cells and its mammospheres but also failed to show any significant difference between these two cell types (Figure 5D). Cumulatively, these results validate that the higher pro-migratory milieu in bCSCs results from greater transcriptional activity of β-catenin.

### Activation of β-catenin/Slug pathway hinders expression of E-cadherin in bCSCs

It is reported that both the EMT-promoting transcription factors, Slug and Snail, the transcriptional target genes of β-catenin, are potent transcriptional repressors of the E-cadherin gene [32]. Our results above, showing significantly greater *Slug* gene expression in mammospheres than in MCF-7 cells with very low expression levels of Snail in both of the cell types, tempted us to evaluate whether the repression of E-cadherin in bCSCs was mediated through the β-catenin/Slug pathway. To that end, siRNA-mediated silencing of Slug in mammospheres resulted in restoration of E-cadherin expression at both protein and mRNA levels (Figure 5E). Under such conditions, the migration potential of the mammospheres was simultaneously retarded as was assessed by monitoring (i) adhesion, that is, 52.67% ± 5.61% cells adhered in Slug-silenced mammospheres as compared with the control set (100%, *P* <0.01) (Figure 5F); (ii) spreading, that is, 0.49 ± 0.03 and 0.7 ± 0.04 mean circularity in control and Slug-ablated mammospheres, respectively (*P* <0.05; Figure 5G, *left panel*); (iii) invasion, that is, 46.67% ± 4.05% invasion in Slug-siRNA-transfected mammospheres as compared with control, that is, (100%, *P* <0.001) (Figure 5G, *middle panel*); and (iv) transwell migration, that is, 37.33% ± 5.04% in Slug knocked-down mammospheres as compared with 100% migration of the control (*P* <0.001; Figure 5G, *right panel*) of MCF-7 cells. The effect of Slug silencing in migration potential was further validated in mammospheres of T47D cells (28% ± 5.69% migration as compared with control, *P* <0.001, Figure 5G, *right panel*). All of these results confirmed that E-cadherin repression in bCSCs results from the activation of the β-catenin/Slug pathway.

### Curcumin abrogates bCSC migration in a dose-dependent manner by restoring EMT-suppressor, E-cadherin

**Figure 6:**
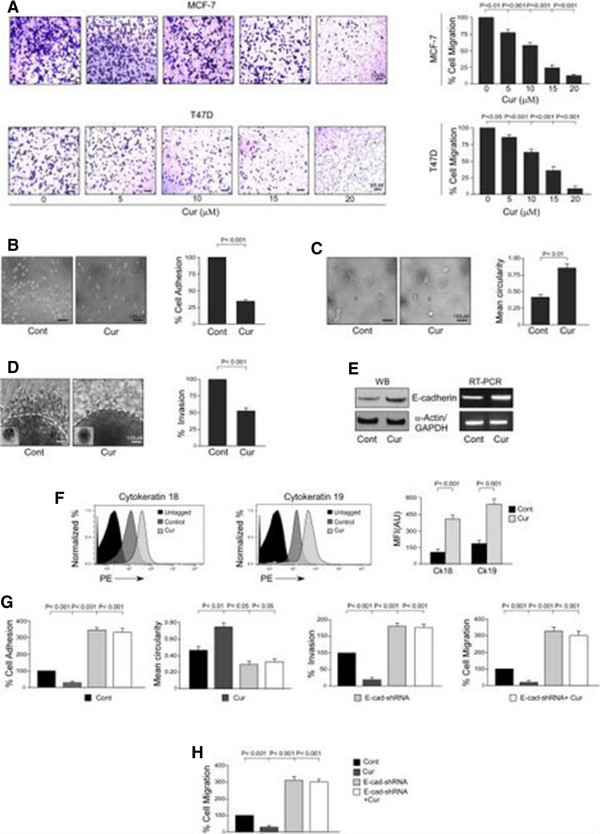
**Curcumin abrogates breast cancer stem cell migration by restoration of E-cadherin expression. (A)** Graphical illustration of transwell migration assay performed to study the effects of 24-hour curcumin treatment on migration of 2° mammosphere-forming cells of MCF-7 and T47D cell lines in a dose-dependent manner (right panels). Representative brightfield images are provided in the left panels. **(B-D)** Representative phase-contrast images of cell adhesion, spreading, and three-dimensional (3D) invasion assays of 2° mammospheres of MCF-7 cell line with or without curcumin treatment (left panels) along with their graphical quantifications (right panels). **(E)** Protein and mRNA expression profiles of E-cadherin in 2° mammospheres of MCF-7 cell line with or without curcumin treatment were determined by Western-blotting (left panel) and reverse transcription-polymerase chain reaction (RT-PCR) (right panel). **(F)** Graphical representation of relative mean fluorescence intensities (MFIs) in arbitrary units (AU) of epithelial markers cytokeratins 18 and 19 in 2° mammospheres of MCF-7 cell line with or without curcumin treatment were determined by flow cytometry and represented graphically (right panel). The left panel depicts representative flow cytometric histogram overlay data. **(G)** Graphical quantifications of cell adhesion, spreading, 3D invasion, and transwell migration assays of MCF-7-derived 2° mammospheres with or without curcumin and E-cadherin-short hairpin RNA (shRNA) treatment/transfection. **(H)** Transwell migration assay was performed under similar experimental conditions as mentioned above in T47D-derived 2° mammospheres. α-Actin/glyceraldehyde 3-phosphate dehydrogenase (GAPDH) was used as an internal loading control. Data are presented as mean ± standard error of mean or representative of three independent experiments. Cont, control; Cur, curcumin.

The phytochemical curcumin is a known repressor of several tumor properties, including tumor cell migration [18]. Additionally, several recent studies suggest that CSCs could be targeted by using curcumin [33–35]. However, there are no detailed studies on the anti-migratory role of curcumin in CSCs. Results of our transwell migration assay revealed that 24-hour curcumin treatment inhibits migration of bCSC-enriched mammospheres of both MCF-7 and T47D cells in a dose-dependent manner (Figure 6A). Our cell viability assay data showed that curcumin exerted apoptotic effects on mammospheres of both MCF-7 and T47D cells beyond a 15 μM dose (Additional file 1: Figure S1). Therefore, to avoid the possibility of curcumin-induced cell death in our experimental set-up, further experiments were restricted to the 15 μM dose of this phytochemical. Additional validation of the effects of curcumin on adhesion, spreading, and 3D invasion properties of mammospheres—that is, 26% ± 3.46% cell adhesion, *P* <0.001 (Figure 6B) and 44% ± 4.36% invasion, *P* <0.001 (Figure 6D) as compared with 100% value of the respective control sets, and 0.46 ± 0.02 and 0.80 ± 0.05 mean circularity (Figure 6C) in control and curcumin-treated mammospheres, respectively (*P* <0.01)—confirmed the anti-migratory effects of curcumin on bCSCs. In fact, curcumin treatment resulted in upregulation of E-cadherin expression at both protein and mRNA levels in mammosphere-forming cells (Figure 6E). To find out whether curcumin exposure altered only E-cadherin expression or overall epithelial characteristics of these bCSCs, flow cytometric analyses of other epithelial markers cytokeratin-18 and -19 were performed. The results revealed that curcumin augmented the overall epithelial characteristics of these cells (Figure 6F). On the other hand, silencing E-cadherin expression by using shRNA significantly nullified the effects of curcumin on the various migratory phenotypes of these CSCs, namely, cell adhesion (351.67% ± 10.14%), 3D invasion (174% ± 7.37%), and migration (304.67% ± 23.79%), as compared with the value of 100% of the respective control sets (*P* <0.001 in each case) (Figure 6G). The results of mean circularity of control (0.463 ± 0.03) and E-cadherin shRNA-transfected mammospheres (0.276 ± 0.03) of MCF-7 cells (*P* <0.05; Figure 6G) were in line with these findings that silencing E-cadherin expression significantly nullified the effects of curcumin on various migratory phenotypes of these CSCs. These results were validated in T47D cells in which higher migration of E-cadherin shRNA-transfected cells of mammospheres (281.67% ± 14.81%) was observed in comparison with untransfected ones (100%, *P* <0.001; Figure 6H). These results together indicated that curcumin inhibited bCSC migration property by restoration of the EMT-suppressor, E-cadherin.

### Curcumin retards activation of β-catenin/Slug pathway in bCSCs thereby restoring E-cadherin

**Figure 7:**
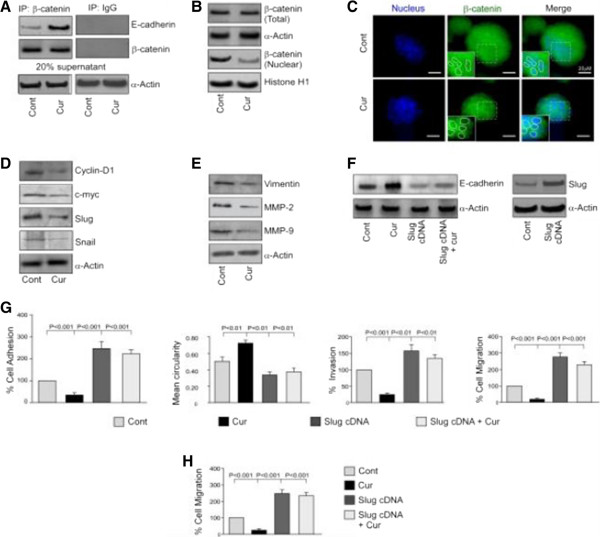
**Curcumin retards activation of** **β-catenin/Slug pathway in breast cancer stem cells, thereby restoring E-cadherin. (A)** β-catenin-associated E-cadherin was assayed by co-immunoprecipitation from cell lysates of MCF-7-derived 2° mammospheres with or without curcumin treatment using specific antibodies (left panel) or with normal human immunoglobulin G (IgG) as a negative control (right panel). To ensure comparable protein loading, 20% of supernatant from immunoprecipitation (IP) sample was subjected to determination of α-actin by Western blotting. **(B)** Western blotting was conducted to study the levels of total β-catenin and nuclear β-catenin in 2° mammospheres in presence or absence of curcumin exposure. **(C)** The relative nuclear expression of β-catenin in 2° mammospheres with or without curcumin treatment was visualized by immunofluorescence. **(D, E)** Under similar conditions, Western blotting was performed to study the expression levels of β-catenin target genes *Cyclin-D1, c-Myc, Slug, Snail, Vimentin, MMP-2 and MMP-9* in curcumin treated/untreated 2° mammospheres. **(F)** Protein expression of E-cadherin in 2° mammospheres with or without curcumin or Slug-cDNA (or both) were determined by Western blotting (left panel). The efficiency of transfection was determined by Western blot analysis (right panel). **(G)** Graphical quantifications of cell adhesion, spreading, three-dimensional (3D) invasion, and migration assays of MCF-7-derived 2° mammospheres with or without curcumin and Slug-cDNA treatment/transfection. **(H)** Transwell migration assay was performed under similar experimental conditions in T47D-derived 2° mammospheres. α-Actin/histone H1/glyceraldehyde 3-phosphate dehydrogenase (GAPDH) was used as an internal loading control. Data are presented as mean ± standard error of mean or representative of three independent experiments. Cont, control; Cur, curcumin.

Since curcumin is reported to be a repressor of β-catenin activity [36], we next attempted to explore whether curcumin could tamper the functioning of β-catenin in bCSCs. Our co-immunoprecipitation studies revealed that curcumin treatment results in significant elevation of E-cadherin/β-catenin complex formation in mammospheres (Figure 7A). A further search for the underlying reason exposed inhibition of the nuclear translocation of β-catenin protein upon curcumin treatment, although the phytochemical failed to alter the total protein level of β-catenin (Figure 7B). This result was confirmed by confocal microscopy (Figure 7C). That curcumin treatment diminished the transcriptional function of β-catenin was evident from our Western blot data in which curcumin-treated mammospheres expressed lower protein levels of its transcriptional targets cyclin D1, c-myc, and Slug, whereas no detectable change was observed in case of Snail, the expression level of which was already very low (Figure 7D). Furthermore, our Western blot data depicted curcumin-mediated downregulation of other β-catenin-induced EMT-promoting factors like vimentin [37] and MMP-2 and MMP-9 [12] in mammospheres (Figure 7E).Curcumin-induced inhibition in Slug expression tempted us to verify whether curcumin-mediated restoration of E-cadherin expression was due to the suppression of the β-catenin/Slug pathway. Our results indicated that transfection of mammosphere-forming cells with Slug overexpression clone significantly downregulated curcumin effect on E-cadherin protein expression (Figure 7F) and considerably nullified curcumin-mediated abrogation of MCF-7-derived bCSC migration as evident from our adhesion, spreading, 3D invasion, and migration assays (Figure 7G). These results were confirmed in T47D-derived 2° mammospheres by performing transwell migration assay under conditions similar to those mentioned above (Figure 7H). Taken together, these results establish that curcumin abrogates migration of bCSCs through perturbation of β-catenin/Slug pathway and restoration of E-cadherin.

**Figure 8:**
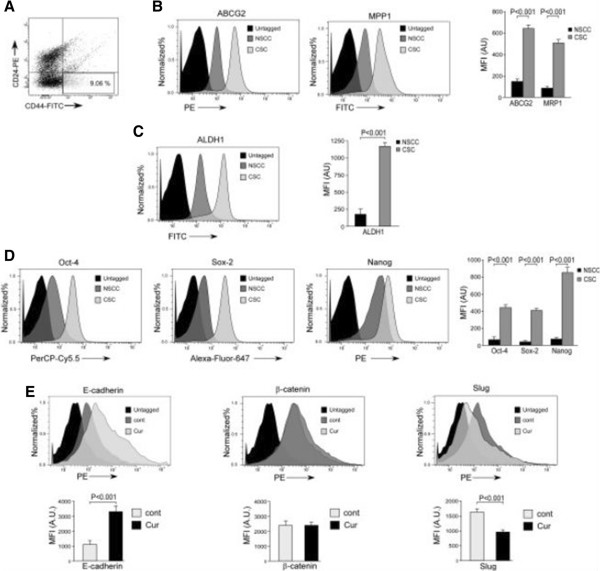
***In vitro*** **validation of the effects of curcumin on primary tumor-derived breast cancer stem cells (bCSCs). (A)** Representative flow cytometric cell-sorting data performed for the purification of bCSCs (CD44^+^/CD24^-/low^) from patient-derived primary breast tumor samples. **(B-D)** Graphical representation of relative mean fluorescence intensities (MFIs) in arbitrary units (AU) of drug-resistance markers ABCG2 and MRP1, stemness related enzyme ALDH1, and de-differentiation markers Oct-4, Sox-2, and Nanog in bCSCs and non-stem cancer cells (NSCCs) purified from primary breast tumors, as determined by flow cytometry (right panels). Left panels depict representative flow cytometric histogram overlay data. **(E)** Graphical representation of relative MFIs of E-cadherin, β-catenin and Slug in the bCSC population of primary tumor samples with or without curcumin treatment as determined by flow cytometry (lower panels). Upper panels depict representative flow cytometric histogramoverlay data. Data are presented as mean ± standard error of mean or representative of three independent experiments. Cont, control; CSC, cancer stem cell; Cur, curcumin.

We next validated our *in vitro* results in bCSCs of primary breast tumor samples. For this purpose, we purified bCSCs flow-cytometrically from primary breast tumors on the basis of the cell surface phenotype CD44^+^/CD24^-/low^ (Figure 8A) and confirmed their stemness properties on the basis of the expression levels of the following markers: MRP1 and ABCG2 (Figure 8B), ALDH1 (Figure 8C), and Oct-4, Sox-2, and Nanog (Figure 8D). These primary tumor-derived bCSCs were next treated with curcumin for further validation of our above-mentioned *in vitro* findings. In fact, supporting our *in vitro* data, curcumin treatment rendered an increase in E-cadherin protein expression along with a concurrent decrease in the expression level of Slug in breast tumor bCSCs, although no significant change in the expression of β-catenin protein was observed (Figure 8E).

Our findings altogether establish a novel role of curcumin in abrogating bCSC migration by restoring the expression of the EMT suppressor E-cadherin, which is normally suppressed in these highly migratory cells, *via* amplification of E-cadherin/β-catenin negative feedback loop.

## Discussion

In the present study, we have disclosed the inhibiting effect of the phytochemical curcumin on migration property of bCSCs. Curcumin is reportedly a potent anti-cancer agent which exerts its anti-cancer effects in multiple ways, including negative regulation of cancer metastasis, angiogenesis, apoptosis, cell-cycle progression, inflammation, and multi-drug resistance [24, 25, 38, 39]. In addition, curcumin can target resistant CSCs by inducing apoptosis [40]. In this study, we assessed the highly aggravated migration potential of bCSCs as compared with the non-stem tumor cells, which comprise the vast majority of the tumor. Aggressiveness of CSCs may justify the gradual increase in the CSC content of the primary tumor with increasing aggressiveness. Interestingly, curcumin retarded the migration potential of the bCSCs by restoring the expression of the inherently repressed EMT-suppressor, E-cadherin. An in-depth study revealed that the curcumin effect was materialized by the downregulation of nuclear β-catenin expression, which in turn negatively affected its pro-migratory transcriptional role, resulting in the diminished expression of its target gene *Slug*, a transcriptional repressor of E-cadherin. From another view point, curcumin treatment rendered greater E-cadherin/β-catenin complex formation which further perturbed the nuclear import of β-catenin. Thus, our work elucidates a thus-far-unknown novel role of curcumin in targeting the aggravated migration property of bCSCs by amplifying the E-cadherin/β-catenin negative feedback loop.

According to the recent studies, CSCs not only are tumor initiators but also are culprits of tumor metastasis [41]. Importantly, these CSCs are largely evasive of the currently existing cancer treatment modalities [42]. This major drawback of existing treatment strategies has led to the growth of considerable interest in alternative treatments using natural remedies since they are more tolerated and cause no significant toxicity as compared with the side effects of chemotherapeutic treatments. Curcumin, the Indian spice derived from the rhizomes of turmeric (*Curcuma longa*), has been intensively studied as a cancer chemopreventive agent in a wide range of cancer models [38, 39, 43]. A plethora of molecular targets and signaling pathways have been shown to be modulated by curcumin, resulting in inhibition of cancer cell proliferation, invasion, metastasis, angiogenesis, and induction of apoptosis [38, 39]. In recent years, curcumin has also been reported to exert its anti-cancer effects on CSCs [40]. A very recent report demonstrated that curcumin targets breast cancer stem-like cells with micro-tentacles that persist in mammospheres and promote reattachment [44]. However, there are no detail studies on the anti-migratory effects of curcumin on CSCs.

Metastasis is responsible for more than 90% of cancer-associated mortality, thereby justifying the clinical need to prevent or target metastasis [45, 46]. It is known that EMT is an essential developmental process that enables reprogramming of polarized epithelial cells toward a mesenchymal motile phenotype [47]. In normal adult tissues, the typically dormant EMT program is reactivated during wound repair and tissue regeneration. In contrast, the non-metastatic cancer cells may undergo the EMT program to attain the migratory and invasive potential required for metastatic progression [48]. Recently, gene expression patterns in human cancers have indicated that de-differentiated cancer cells combine EMT properties with a stem-cell like phenotype [11, 49]. A direct molecular link between EMT and stemness was demonstrated by the observations that EMT activators, such as transforming growth factor-beta 1 (TGFβ1), can co-induce EMT and stemness properties, thereby linking the EMT and CSC concept [50]. Cancer cells that undergo EMT lose epithelial polarity and acquire invasive properties and stem cell-like features, which are believed to prelude metastasis [51]. This correlation between EMT and CSCs suggests that the treatment strategies which target EMT regimen may virtually restrain both CSCs and metastatic potential of the tumor. In our present study, we report a novel function of curcumin in disrupting this complex ‘EMT-ambience’ of bCSCs which is also responsible for their aggravated migratory property. Therefore, our results are in keeping with a recent concept suggesting that CSCs have characteristics of post-EMT cancer cells and that these features further aid these CSCs for successful metastatic colonization [41]. Targeting this transition (EMT) among CSCs by curcumin therefore opens a new arena for the treatment of highly malignant breast cancers.

Loss of E-cadherin-mediated cell adhesion is one of the key mechanisms involved in metastatic conversion of epithelial cells and EMT [52]. Numerous studies have described a partial or complete loss of E-cadherin during cancer progression, which is often correlated with an unfavorable prognosis [53], confirming E-cadherin to be a caretaker of the epithelial state. One of the probable mechanisms involved in E-cadherin dysfunction, especially loss of its expression and consequent promotion of tumor progression, is through β-catenin signaling [54]. As the main binding partner of β-catenin, E-cadherin plays a pivotal role in β-catenin stabilization and function. Their functional complex formation is necessary for adhesion and the maintenance of epithelial cell layers. The loss of E-cadherin results in nuclear localization of endogenous β-catenin [55]. On the other hand, E-cadherin could potentially antagonize signaling activity of β-catenin by sequestering β-catenin at the membrane, thus keeping it away from the nucleus, as well as by competing with LEF-1 for the same binding sites on β-catenin. If E-cadherin fails to associate with β-catenin, E-cadherin is retained in the endoplasmic reticulum, where it is subsequently degraded [56]. A functional cadherin-catenin complex is therefore important for maintaining cellular integrity [57]. In fact, increased concentration of free β-catenin in the cytoplasm promotes its binding to the LEF/TCF family of DNA-binding proteins. As a result, β-catenin translocates to the nucleus, where it transcriptionally activates specific target genes [12]. Accumulating evidence suggests that several transcription factors, including Slug and Snail, both of which are transcriptional targets of β-catenin, are implicated in E-cadherin repression and EMT [12]. However, the expression of Snail is suppressed in estrogen receptor-positive (ER^+^) breast cancer cell lines like MCF-7 and T47D [58], whereas Slug has been proposed to be a likely *in vivo* repressor of E-cadherin as compared with Snail in breast carcinomas [59]. A report from Hajra *et al.*[59] further demonstrated that, in breast cancer cell lines, Slug expression correlates more strongly than snail expression with E-cadherin suppression [59]. In keeping with these reports, our results also revealed low expression of Snail in the ER^+^ breast cancer cell line, MCF-7, while Slug played the key role in maintaining the aggravated migration potential of bCSCs.

In breast cancer, curcumin has also been shown to repress expression of Slug [60]. More recently, a report on MCF-7 cells showed that the effect of curcumin on bCSCs is mediated through the inhibition of the self-renewal property of these cells by exerting potent inhibition on Wnt/β-catenin signaling [33]. Here, we report the yet-unexplored anti-migratory effect of curcumin on bCSCs by restoration of E-cadherin expression, which is mediated through the negative regulation of the β-catenin/Slug pathway. In fact, curcumin restores the E-cadherin/β-catenin complex formation, which further impedes nuclear transport of β-catenin that in turn increases E-cadherin expression through inhibition of Slug, thereby accentuating the E-cadherin/β-catenin negative feedback loop. Cumulatively, curcumin targets bCSC migration by suppressing their EMT phenotype, which in general imparts higher migration potential to tumor cells.

## Conclusions

We demonstrate for the first time, to the best of our knowledge, that the phytochemical curcumin inhibits the aggravated migration potential of bCSCs by perturbing commencement of EMT. This could be a very promising addition to traditional cancer treatments, including chemotherapy and radiotherapy, especially given the fact that most chemotherapeutic drugs and radiotherapy do not have the capability to eliminate CSCs. This combination strategy will open a new avenue for more effective therapies for highly invasive breast carcinomas.

## Electronic supplementary material

Additional file 1: Figure S1: (A and B) Effects of dose-dependent treatment of curcumin on the percent cell viability of 2° spheres derived from MCF-7 and T47D cells. Data are presented as mean ± SEM or representative of three independent experiments. **p < 0.01. (JPEG 167 KB)

## Abbreviations

3D: three-dimensional
ALDH1: aldehyde dehydrogenase isoform 1
bCSC: breast cancer stem cell
BSA: bovine serum albumin
CSC: cancer stem cell
DMEM: Dulbecco’s modified Eagle’s medium
EMT: epithelial-mesenchymal transition
ER: estrogen receptor
FAK: focal adhesion kinase
FBS: fetal bovine serum
FITC: fluorescein isothiocyanate
LEF: lymphoid enhancer factor
MMP: matrix metalloproteinase
PBS: phosphate-buffered saline
PCR: polymerase chain reaction
PE: phycoerythin
shRNA: short hairpin RNA
siRNA: small interfering RNA
TCF: T-cell factor.

## References

[1] Zhang Z, Ni C, Chen W, Wu P, Wang Z, Yin J, Huang J, Qiu F (2014). Expression of CXCR4 and breast cancer prognosis: a systematic review and meta-analysis. BMC Cancer.

[2] Minn AJ, Kang Y, Serganova I, Gupta GP, Giri DD, Doubrovin M, Ponomarev V, Gerald WL, Blasberg R, Massagué J (2005). Distinct organ-specific metastatic potential of individual breast cancer cells and primary tumors. J Clin Invest.

[3] Malanchi I, Santamaria-Martínez A, Susanto E, Peng H, Lehr HA, Delaloye JF, Huelsken J (2011). Interactions between cancer stem cells and their niche govern metastatic colonization. Nature.

[4] Al-Hajj M, Clarke MF (2004). Self-renewal and solid tumor stem cells. Oncogene.

[5] Armstrong AJ, Marengo MS, Oltean S, Kemeny G, Bitting RL, Turnbull JD, Herold CI, Marcom PK, George DJ, Garcia-Blanco MA (2011). Circulating tumor cells from patients with advanced prostate and breast cancer display both epithelial and mesenchymal markers. Mol Cancer Res.

[6] van der Horst G, Bos L, van der Pluijm G (2012). Epithelial plasticity, cancer stem cells, and the tumor-supportive stroma in bladder carcinoma. Mol Cancer Res.

[7] Malanchi I (2013). Tumour cells coerce host tissue to cancer spread. Bonekey Rep.

[8] Uchino M, Kojima H, Wada K, Imada M, Onoda F, Satofuka H, Utsugi T, Murakami Y (2010). Nuclear beta-catenin and CD44 upregulation characterize invasive cell populations in non-aggressive MCF-7 breast cancer cells. BMC Cancer.

[9] Xiong H, Hong J, Du W, Lin YW, Ren LL, Wang YC, Su WY, Wang JL, Cui Y, Wang ZH, Fang JY (2012). Roles of STAT3 and ZEB1 proteins in E-cadherin downregulation and human colorectal cancer epithelial-mesenchymal transition. J Biol Chem.

[10] van de Wetering M, de Lau W, Clevers H (2002). WNT signaling and lymphocyte development. Cell.

[11] Brabletz T, Jung A, Spaderna S, Hlubek F, Kirchner T (2005). Migrating cancer stem cells - an integrated concept of malignant tumor progression. Nat Rev Cancer.

[12] Thakur R, Mishra DP (2013). Pharmacological modulation of beta-catenin and its applications in cancer therapy. J Cell Mol Med.

[13] Martin TA, Goyal A, Watkins G, Jiang WG (2005). Expression of the transcription factors snail, slug, and twist and their clinical significance in human breast cancer. Ann Surg Oncol.

[14] De Craene B, Gilbert B, Stove C, Bruyneel E, van Roy F, Berx G (2005). The transcription factor snail induces tumor cell invasion through modulation of the epithelial cell differentiation program. Cancer Res.

[15] Zhang YG, DU J, Tian XX, Zhong YF, Fang WG (2007). Expression of E-cadherin, beta-catenin, cathepsin D, gelatinases and their inhibitors in invasive ductal breast carcinomas. Chin Med J (Engl).

[16] Savagner P, Yamada KM, Thiery JP (1997). The zinc-finger protein slug causes desmosome dissociation, an initial and necessary step for growth factor-induced epithelial-mesenchymal transition. J Cell Biol.

[17] Ouhtit A, Gaur RL, Abdraboh M, Ireland SK, Rao PN, Raj SG, Al-Riyami H, Shanmuganathan S, Gupta I, Murthy SN, Hollenbach A, Raj MH (2013). Simultaneous inhibition of cell-cycle, proliferation, survival, metastatic pathways and induction of apoptosis in breast cancer cells by a phytochemical super-cocktail: genes that underpin its mode of action. J Cancer Educ.

[18] Kunnumakkara AB, Anand P, Aggarwal BB (2008). Curcumin inhibits proliferation, invasion, angiogenesis and metastasis of different cancers through interaction with multiple cell signaling proteins. Cancer Lett.

[19] Kakarala M, Brenner DE, Korkaya H, Cheng C, Tazi K, Ginestier C, Liu S, Dontu G, Wicha MS (2010). Targeting breast stem cells with the cancer preventive compounds curcumin and piperine. Breast Cancer Res Treat.

[20] Saha S, Hossain DM, Mukherjee S, Mohanty S, Mazumdar M, Mukherjee S, Ghosh UK, Nayek C, Raveendar C, Khurana A, Chakrabarty R, Sa G, Das T (2013). Calcarea carbonica induces apoptosis in cancer cells in p53-dependent manner via an immuno-modulatory circuit. BMC Complement Altern Med.

[21] Mohanty S, Saha S, Md S, Hossain D, Adhikary A, Mukherjee S, Manna A, Chakraborty S, Mazumdar M, Ray P, Das K, Chakraborty J, Sa G, Das T (2014). ROS PIASγ cross talk channelizes ATM signaling from resistance to apoptosis during chemosensitization of resistant tumors. Cell Death Dis.

[22] Dontu G, Abdallah WM, Foley JM, Jackson KW, Clarke MF, Kawamura MJ, Wicha MS (2003). In vitro propagation and transcriptional profiling of human mammary stem/progenitor cells. Genes Dev.

[23] Hossain DM, Panda AK, Manna A, Mohanty S, Bhattacharjee P, Bhattacharyya S, Saha T, Chakraborty S, Kar RK, Das T, Chatterjee S, Sa G (2013). FoxP3 acts as a cotranscription factor with STAT3 in tumor-induced regulatory T cells. Immunity.

[24] Bhattacharyya S, Mandal D, Saha B, Sen GS, Das T, Sa G (2007). Curcumin prevents tumor-induced T cell apoptosis through Stat-5a-mediated Bcl-2 induction. J Biol Chem.

[25] Choudhuri T, Pal S, Das T, Sa G (2005). Curcumin selectively induces apoptosis in deregulated cyclin D1-expressed cells at G2 phase of cell cycle in a p53-dependent manner. J Biol Chem.

[26] Mazumdar M, Adhikary A, Chakraborty S, Mukherjee S, Manna A, Saha S, Mohanty S, Dutta A, Bhattacharjee P, Ray P, Chattopadhyay S, Banerjee S, Chakraborty J, Ray AK, Sa G, Das T (2013). Targeting RET to induce medullary thyroid cancer cell apoptosis: an antagonistic interplay between PI3K/Akt and p38MAPK/caspase-8 pathways. Apoptosis.

[27] Croker AK, Goodale D, Chu J, Postenka C, Hedley BD, Hess DA, Allan AL (2009). High aldehyde dehydrogenase and expression of cancer stem cell markers selects for breast cancer cells with enhanced malignant and metastatic ability. J Cell Mol Med.

[28] Shin SY, Rath O, Zebisch A, Choo SM, Kolch W, Cho KH (2010). Functional roles of multiple feedback loops in extracellular signal-regulated kinase and Wnt signaling pathways that regulate epithelial-mesenchymal transition. Cancer Res.

[29] Hlubek F, Spaderna S, Jung A, Kirchner T, Brabletz T (2004). Beta-catenin activates a coordinated expression of the proinvasive factors laminin-5 gamma2 chain and MT1-MMP in colorectal carcinomas. Int J Cancer.

[30] Chen L, Li M, Li Q, Wang CJ, Xie SQ (2013). DKK1 promotes hepatocellular carcinoma cell migration and invasion through β-catenin/MMP7 signaling pathway. Mol Cancer.

[31] Mao J, Fan S, Ma W, Fan P, Wang B, Zhang J, Wang H, Tang B, Zhang Q, Yu X, Wang L, Song B, Li L (2014). Roles of Wnt/β-catenin signaling in the gastric cancer stem cells proliferation and salinomycin treatment. Cell Death Dis.

[32] Heuberger J, Birchmeier W (2010). Interplay of cadherin-mediated cell adhesion and canonical Wnt signaling. Cold Spring Harb Perspect Biol.

[33] Li Y, Zhang T (2014). Targeting cancer stem cells by curcumin and clinical applications. Cancer Lett.

[34] Almanaa TN, Geusz ME, Jamasbi RJ (2012). Effects of curcumin on stem-like cells in human esophageal squamous carcinoma cell lines. BMC Complement Altern Med.

[35] Norris L, Karmokar A, Howells L, Steward WP, Gescher A, Brown K (2013). The role of cancer stem cells in the anti-carcinogenicity of curcumin. Mol Nutr Food Res.

[36] Sundram V, Chauhan SC, Ebeling M, Jaggi M (2012). Curcumin attenuates β-catenin signaling in prostate cancer cells through activation of protein kinase D1. PLoS One.

[37] Gilles C, Polette M, Mestdagt M, Nawrocki-Raby B, Ruggeri P, Birembaut P, Foidart JM (2003). Transactivation of vimentin by beta-catenin in human breast cancer cells. Cancer Res.

[38] Sa G, Das T (2008). Anti-cancer effects of curcumin: cycle of life and death. Cell Div.

[39] Saha S, Adhikary A, Bhattacharyya P, DAS T, Sa G (2012). Death by design: where curcumin sensitizes drug-resistant tumours. Anticancer Res.

[40] Lin L, Liu Y, Li H, Li PK, Fuchs J, Shibata H, Iwabuchi Y, Lin J (2011). Targeting colon cancer stem cells using a new curcumin analogue, GO-Y030. Br J Cancer.

[41] Oskarsson T, Batlle E, Massagué J (2014). Metastatic Stem Cells: Sources, Niches, and Vital Pathways. Cell Stem Cell.

[42] Kubo T, Takigawa N, Osawa M, Harada D, Ninomiya T, Ochi N, Ichihara E, Yamane H, Tanimoto M, Kiura K (2013). Subpopulation of small-cell lung cancer cells expressing CD133 and CD87 show resistance to chemotherapy. Cancer Sci.

[43] Bhattacharyya S, Mandal D, Sen GS, Pal S, Banerjee S, Lahiry L, Finke JH, Tannenbaum CS, Das T, Sa G (2007). Tumor-induced oxidative stress perturbs nuclear factor-kappaB activity-augmenting tumor necrosis factor-alpha-mediated T-cell death: protection by curcumin. Cancer Res.

[44] Charpentier MS, Whipple RA, Vitolo MI, Boggs AE, Slovic J, Thompson KN, Bhandary L, Martin SS (2014). Curcumin targets breast cancer stem-like cells with microtentacles that persist in mammospheres and promote reattachment. Cancer Res.

[45] Mehlen P, Puisieux A (2006). Metastasis: a question of life or death. Nat Rev Cancer.

[46] Nguyen DX, Massagué J (2007). Genetic determinants of cancer metastasis. Nat Rev Genet.

[47] May CD, Sphyris N, Evans KW, Werden SJ, Guo W, Mani SA (2011). Epithelial-mesenchymal transition and cancer stem cells: a dangerously dynamic duo in breast cancer progression. Breast Cancer Res.

[48] Thiery JP, Acloque H, Huang RY, Nieto MA (2009). Epithelial-mesenchymal transitions in development and disease. Cell.

[49] Polyak K, Weinberg RA (2009). Transitions between epithelial and mesenchymal states: acquisition of malignant and stem cell traits. Nat Rev Cancer.

[50] Dalerba P, Cho RW, Clarke MF (2007). Cancer stem cells: models and concepts. Annu Rev Med.

[51] Herreros-Villanueva M, Zhang JS, Koenig A, Abel EV, Smyrk TC, Bamlet WR, de Narvajas AA, Gomez TS, Simeone DM, Bujanda L, Billadeau DD (2013). SOX2 promotes dedifferentiation and imparts stem cell-like features to pancreatic cancer cells. Oncogenesis.

[52] Agiostratidou G, Hulit J, Phillips GR, Hazan RB (2007). Differential cadherin expression: potential markers for epithelial to mesenchymal transformation during tumor progression. J Mammary Gland Biol Neoplasia.

[53] Berx G, Van Roy F (2001). The E-cadherin/catenin complex: an important gatekeeper in breast cancer tumorigenesis and malignant progression. Breast Cancer Res.

[54] Prasad CP, Rath G, Mathur S, Bhatnagar D, Parshad R, Ralhan R (2009). Expression analysis of E-cadherin, Slug and GSK3beta in invasive ductal carcinoma of breast. BMC Cancer.

[55] Yang SZ, Kohno N, Yokoyama A, Kondo K, Hamada H, Hiwada K (2001). Decreased E-cadherin augments beta-catenin nuclear localization: studies in breast cancer cell lines. Int J Oncol.

[56] Chen YT, Stewart DB, Nelson WJ (1999). Coupling assembly of the E-cadherin/beta-catenin complex to efficient endoplasmic reticulum exit and basal-lateral membrane targeting of E-cadherin in polarized MDCK cells. J Cell Biol.

[57] Tian X, Liu Z, Niu B, Zhang J, Tan TK, Lee SR, Zhao Y, Harris DC, Zheng G (2011). E-cadherin/β-catenin complex and the epithelial barrier. J Biomed Biotechnol.

[58] Fujita N, Jaye DL, Kajita M, Geigerman C, Moreno CS, Wade PA (2003). MTA3, a Mi-2/NuRD complex subunit, regulates an invasive growth pathway in breast cancer. Cell.

[59] Hajra KM, Chen DY, Fearon ER (2002). The SLUG zinc-finger protein represses E-cadherin in breast cancer. Cancer Res.

[60] Prasad CP, Rath G, Mathur S, Bhatnagar D, Ralhan R (2009). Potent growth suppressive activity of curcumin in human breast cancer cells: modulation of Wnt/beta-catenin signaling. Chem Biol Interact.

