# Supplementary figures and images for: Curcumin inhibits breast cancer stem cell migration by amplifying the E-cadherin/β-catenin negative feedback loop
Source: Stem Cell Res Ther. 2014 Oct 14;5(5):116. doi: 10.1186/scrt506 (PMC4445824; doi:10.1186/scrt506)

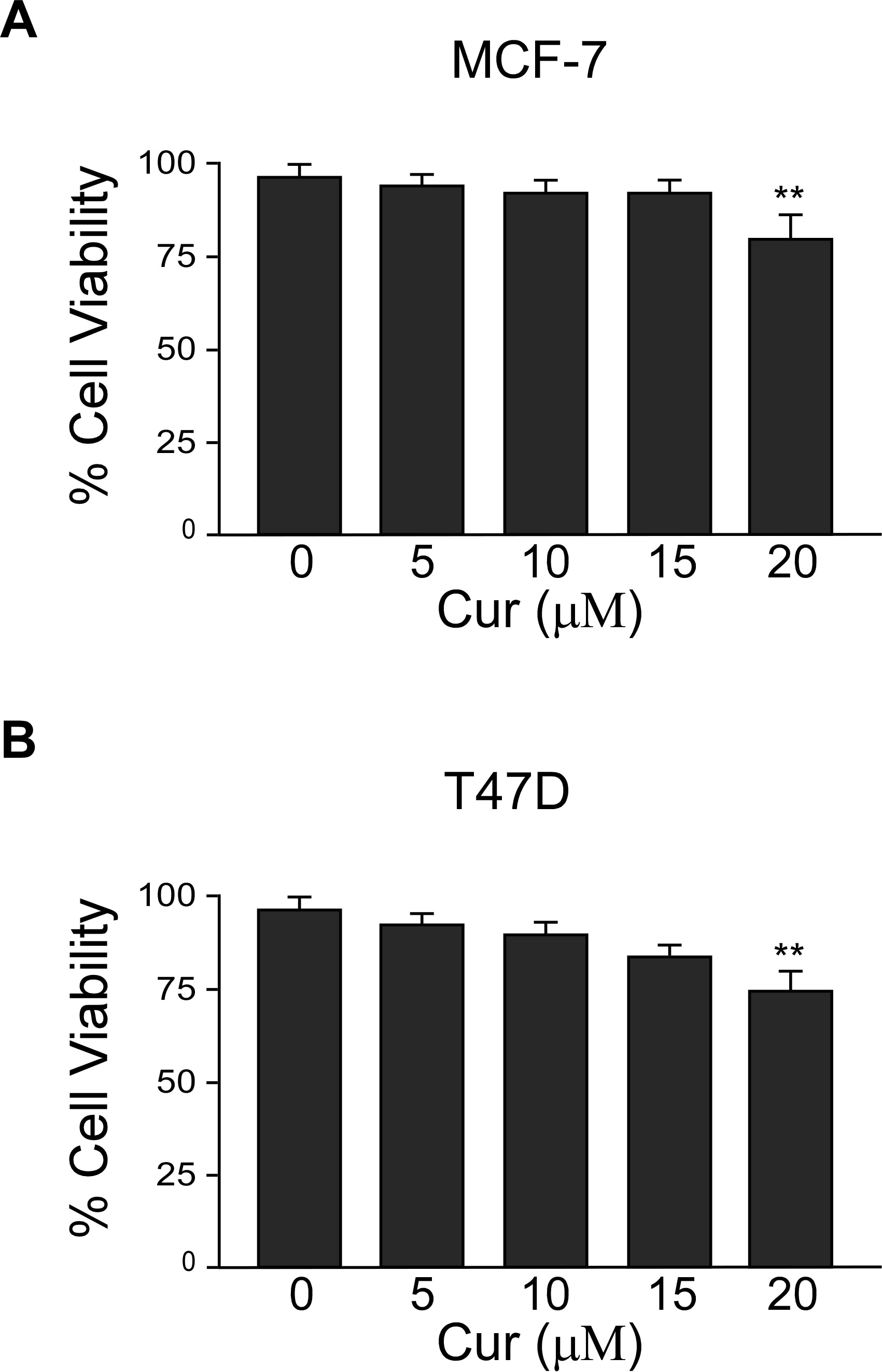

Supplement: Supplementary file 1 — Additional file 1: Figure S1: (A and B) Effects of dose-dependent treatment of curcumin on the percent cell viability of 2° spheres derived from MCF-7 and T47D cells. Data are presented as mean ± SEM or representative of three independent experiments. **p < 0.01. (JPEG 167 KB) [file 13287_2014_430_MOESM1_ESM.jpeg]
